# Hyperhomocysteinemia as a Risk Factor and Potential Nutraceutical Target for Certain Pathologies

**DOI:** 10.3389/fnut.2019.00049

**Published:** 2019-04-24

**Authors:** Caterina Tinelli, Antonella Di Pino, Elena Ficulle, Serena Marcelli, Marco Feligioni

**Affiliations:** ^1^Golgi Cenci Foundation, Abbiategrasso, Italy; ^2^Laboratory of Neuronal Cell Signaling, EBRI Rita Levi-Montalcini Foundation, Rome, Italy; ^3^Laboratory of Neurobiology in Translational Medicine, Department of Neurorehabilitation Sciences, Casa Cura Policlinico, Milan, Italy

**Keywords:** hyperhomocysteinemia, neurological diseases, nutraceuticals, Normocis, cardiovascular, Alzheimer, Parkinson, vitamin

## Abstract

Hyperhomocysteinemia is recognized as a risk factor for several diseases, including cardiovascular and neurological conditions. Homocysteine (HCys) is a key metabolite involved in the biosynthesis and metabolism of methionine (Met), which plays a pivotal role in the physiological cell's life cycle. The biochemistry of Met is finely regulated by several enzymes that control HCys concentration. Indeed, balanced activity among the enzymes is essential for the cell's well-being, while its malfunction could raise HCys concentration which can lead to the onset of several pathological conditions. The HCys concentration increase seems to be caused mainly by the widely diffused polymorphisms of several enzymes. Nowadays, a blood test can easily detect elevated concentrations of HCys, referred to as Hyperhomocysteinemia (HHCys). Prolonged exposure to this condition can lead to the onset of cardiovascular disease and can lead to the development of atherosclerosis, stroke, inflammatory syndromes like osteoporosis and rheumatism, as well as neuronal pathologies including Alzheimer's and Parkinson's diseases. In this review, we analyzed the literature of several pathological conditions in which the molecular pathways of HHCys are involved. Interestingly, several observations indicate that the calibrated assumption of correct doses of vitamins such as folic acid, vitamin B6, vitamin B12, and betaine may control HHCys-related conditions.

## Introduction

A high blood level of Homocysteine (HCys) has been regarded, in the last 10 years, as a biomarker of cardiovascular disease as well as a risk factor for many other pathologies, including Alzheimer's and other dementias (1).

Homocysteine (HCys) is a non-essential amino acid that derives from the biosynthesis and metabolism of methionine (Met). Indeed, within the Met metabolic pathway, HCys either can be irreversibly degraded to cysteine (Cys) via the trans-sulfuration pathway or re-methylated back to Met.

HCys is extremely important for the cell's homeostasis, although its physiological activity is essential to Met, which plays a vital role for the cell's viability.

**Figure 1:**
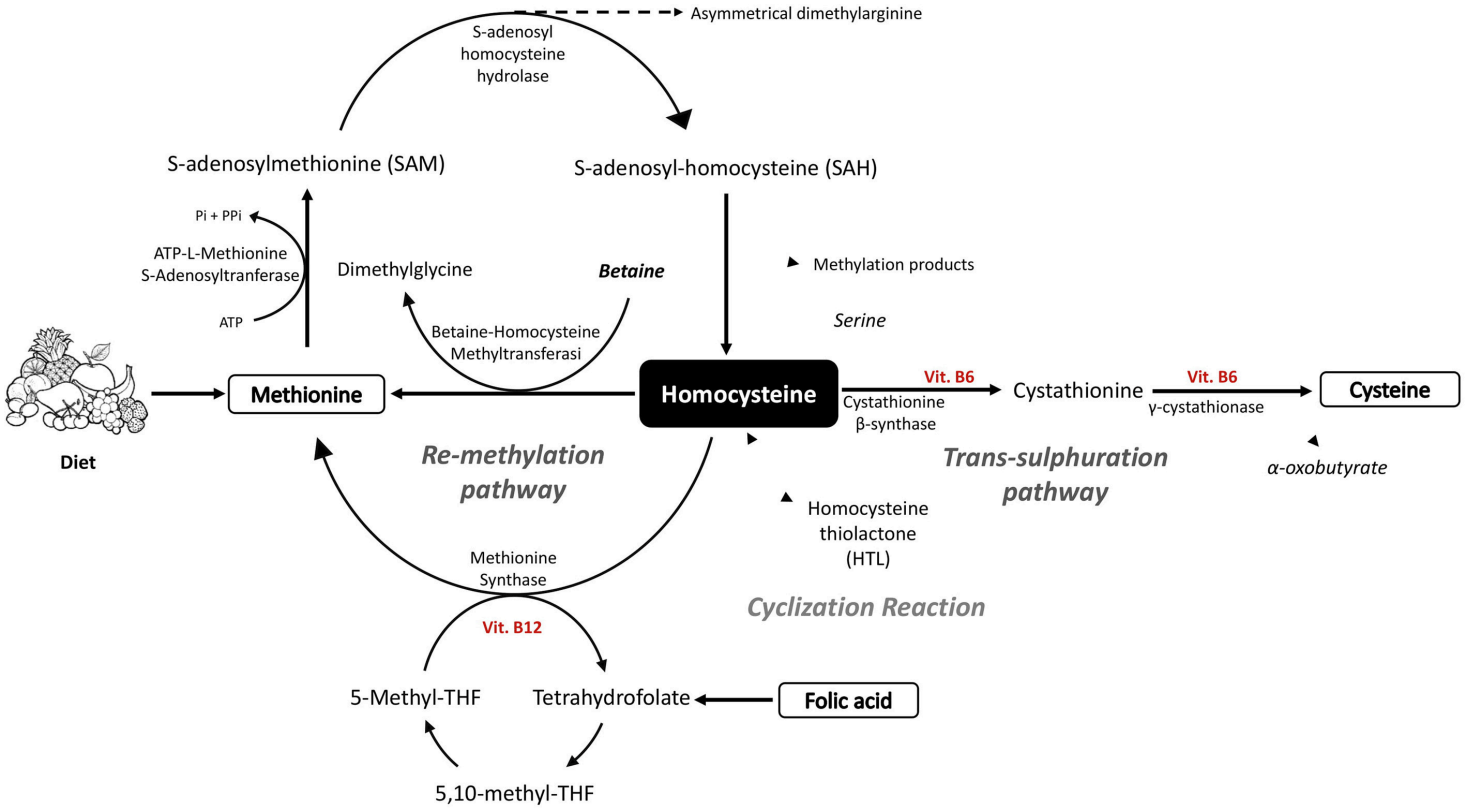
Schematic diagram of homocysteine and methionine biosynthesis, together with the metabolic processes of trans-sulfuration and re-methylation linked to the folate cycle.

Although HCys is not directly involved in protein synthesis, its role in folate metabolism and choline catabolism is fundamental to regulating Met activity. This latter role is, in fact, required for the synthesis of several proteins, in which its ability to donate methyl groups is essential for the synthesis of methylated compounds, while the inorganic sulfate is fundamental for the synthesis of sulfur-containing amino acids. In addition, Met can provide the carbon skeleton for polyamine synthesis (2) (Figure 1).

Although HCys plasma levels can significantly vary among different populations according to their dietary habits, Hyperhomocysteinemia (HHCys) is considered as a biomarker for several pathologies, and therefore the high plasmatic levels of HCys can be easily monitored by blood testing (1, 3).

HCys physiological levels are commonly considered normal between 5 and 15 μmol/l, while HHCys is considered mild when ranging from 15 to 30 μmol/l, intermediate for values between 30 and 100 μmol/l, and serious for values exceeding 100 μmol/l (4).

Persistent HHCys promotes the formation of atherosclerotic plaques, atherothrombotic events through endothelial dysfunction, the enhancement of inflammation and the so-called thrombophilic profile (3). For these reasons, in addition to the traditional risk factors, both the World Health Organization (WHO) and the Health Ministry agreed to consider HHCys, a strong contributor for cardiovascular disease (5).

Elevated concentrations of HCys are indeed implicated in an augmented risk of dementia, in particular Alzheimer's disease, but also in both regional and whole brain atrophy in healthy elderly people (6). Moreover, high levels of HCys were found in the blood of Parkinson's and epileptic patients (7, 8).

The leading causes of HHCys are a range of genetic defects as well as various lifestyle and nutritional habits, in particularly those related to certain pathophysiological conditions and to the use of drugs (3).

The review will show an overview of several pathologies linked to HHCys, pointing out that HHCys should be not only considered as a biomarker and a risk factor for potential pathologies, but should be also mentioned as a nutraceutical target. So far, treatments aiming to decrease homocysteine blood levels, associated with other therapies, have indeed shown a potential effect in preventing stroke episodes (9).

### Biosynthesis and Metabolism of Homocysteine (HCys)

HCys is obtained by a biochemical synthesis from the demethylation of Methionine (Met) and goes through three subsequent steps (10, 11). Firstly, ATP-L-Methionine S-Adenosyltranferase (MAT), by using ATP, transfers to Met the Adenosyl portion, leading to the formation of S-Adenosyl-L-Methionine (AdoMet or SAM). In the second step, the universal methyl donor SAM is involved in the trans-methylation reaction with several acceptors of methyl groups such as DNA, RNA, proteins, and lipids, with a particular involvement in the formation of creatine (12). The resulting compound S-Adenosyl homocysteine (AdoHCys or SAH), lacking the methyl group, is subsequently cleaved to HCys and Adenosine by S-adenosyl homocysteine hydrolase. This reaction is reversible, although the thermodynamic equilibrium favors AdoHCys synthesis since both HCys and adenosine are usually quickly removed, leading the reaction to progress toward hydrolysis (10).

Thus, the HCys so obtained can be metabolized by two reactions, trans-sulfuration or re-methylation (13). The first one routes HCys to the formation of cysteine (Cys) through condensation with serine, obtaining cystathionine as intermediate, which is in turn cleaved into Cys and alpha-oxobutyrate. The reaction of condensation is catalyzed by cystathionine β-synthase (CβS) which needs the cofactor Vitamin B6 to work, and is followed by the catalysis done by γ-cystathionase (11). Alternatively, the methionine synthase (MS), by using vitamin B12 as a cofactor, catalyzes the re-methylation reaction, restoring Met by transferring the methyl group from 5-*N*-methyl tetrahydrofolate (5-methyl-THF) to HCys (11). 5-*N*-methyl-THF is the major source of methyl groups for the re-methylation of HCys although, depending on the body organ, betaine, through the enzyme involved, betaine-homocysteine methyltransferase (BHMT), can also act as methyl group donors. The betaine pathway is mainly restricted to the liver and kidney, in which BHMT is primarily expressed and betaine is an intermediate of choline oxidation (10).

Alternatively, HCys can be erroneously cyclized, leading to the formation of Homocysteine thiolactone (HTL), which is a toxic intermediate. This result is the result of a wrong reaction of methionyl-tRNA synthase, which binds to the HCys rather than to the Met, probably due to the homology in their structure. The biosynthesis mistakes are in any case immediately corrected by leading to the formation of the cyclic intermediate of the HCys (HTL) (14) (Figure 1).

### Metabolism of Folate

Folate, the water-soluble B vitamin, is an essential vitamin contained in fruits and vegetables which is known as coenzyme in nucleic acid synthesis and in Met regeneration (15).

These vitamins are mostly present as polyglutamates which, in order to be absorbed and used by the body, need to be hydrolyzed to monoglutamates. In particular, 5-Methyl-THF, the main form of folate in the plasma, in order to be absorbed by the cells exploits folate receptor α (FR-α), mostly expressed in the proximal tubules of kidneys, the choroid plexus, and the placenta (16).

Two other receptors are known, namely β and γ, that show lower affinity for 5-Methyl-THF in respect to FR-α. Furthermore, a membrane carrier for 5-Methyl-THF known as reduced folate carrier (RFC) is ubiquitously expressed and its affinity is lower than FR-α (17).

Tetrahydrofolate (THF) is an important key player in folate metabolism as folate acceptor molecule. Firstly, THF is converted to 5,10-methylene-THF by the pyridoxal phosphate (PLP)-dependent serine hydroxymethyltransferase (SHMT); later, it is reduced to 5-Methyl-THF by methylene tetrahydrofolate reductase (MTHFR). This intermediate is fundamental since it directly enters into the Met regeneration pathway as methyl donor; in particular, a methyl group is removed by 5-Methyl-THF and transferred firstly to the vitamin B12 coenzyme, and later to HCys.

In fact, around 70% of generated HCys comes from the re-methylation step depending on the content of Met and choline in the diet (18) (Figure 1).

### Catabolism of Choline

Choline is a water-soluble vitamin-like nutrient showing very heterogeneous roles in cell activities. Its chemical structure is present in several molecules such as acetylcholine, phospholipids (phosphatidylcholine, glycerophosphocholine, phosphocholine, and sphingomyelin), and lipoproteins; thereby, choline becomes important for cell structure component and lipid transport, but also for neurotransmitter synthesis and methyl-group metabolism (HCys reduction).

Choline levels depend both on diet intake as well as *de novo* biosynthesis in the methylation of phosphatidylethanolamine (PE) to phosphatidylcholine (PC) (19).

Whenever choline levels become low, liver and brain cells react by recruiting the choline-based molecules from kidney, lung, and intestine (20).

Choline plays a significant role in Met regeneration since, being oxidized to betaine, it can provide the one-carbon unit used in the conversion from homocysteine to methionine (21) (Figure 1).

### Causes of Hyperhomocysteinemia

The major cause of HHCys is the genetic defects of the transcription of enzymes responsible for the HCys metabolism, which has been an object of interest for scientific research (22–25). In particular, the polymorphisms of the main enzymes involved in HCys metabolism such us Methylenetetrahydrofolate reductase (MTHFR), Cystathionine β-synthase, Methionine synthase, Methionine synthetase reductase, and Methionine adenosyltransferase IA, have been identified as interesting subjects of studies (3, 26).

One of the most studied polymorphisms is C677T, present on the gene encoding for the folate-metabolizing enzyme MTHFR. It has been estimated that 10% of the worldwide population is homozygous (TT genotype) for the common C677T polymorphism, but the frequency can rise up to 25% in southern Italy and to 32% in some areas in Mexico. Although the real causes of the high incidences of C677T polymorphism in some areas of the world are still under study, being aware of the prevalence of these polymorphisms in different geographical areas could be helpful for clinical practice. Indeed, the close relationship between MTHFR polymorphisms and folate levels in the serum of the mothers raises a question about the use of dietary supplements containing folic acid by pregnant women.

The TT genotype is responsible for the reduced activity of the MTHFR enzyme, which in turn leads to an increase of HCys concentrations (22). Molecular studies pursued on individuals carrying the TT genotype have shown that mutated MTHFR enzymes have a decreased affinity for riboflavin cofactor (22, 23), which has been recently shown to be an important modulator of HCys concentration, especially in individuals with TT genotype (22, 23, 25).

Another important polymorphism is *T833C* which is present, as a mutation, on the gene encoding for cystathionine β synthase (CβS), an enzyme that takes part in the trans-sulfuration pathway in HCys metabolism converting HCys in cystathionine. The T to C replacement in the 833 nucleotide causes an Ile to Thr amino acid substitution, implying an alteration in CβS activity (27) and increasing HCys levels.

Considering the huge impact of genetic polymorphisms on the HCys level increase, current studies are focused on establishing the correlation between polymorphisms and stroke events (24, 28). Although the results are still conflicting, Ding et al. have shown that several genetic models associated with polymorphisms related to HCys metabolism are susceptible to stroke (24).

In addition to genetic causes, many others depend mainly on habits and lifestyle have been identified as being responsible for HHCys. For example, nutritional deficiencies of some of the cofactors involved in HCys metabolism—such as folic acid, vitamin B6, vitamin B12, and betaine—are undoubtedly responsible for the development of HHCys. Folic acid consumption is reduced especially in those countries in which the fortification of cereal-grain products is absent or rare. For example, it has been reported that 33.8% of preschool-age children in Venezuela contain a folate deficit, compared with 48.8% of pregnant woman in Costa Rica and 25.5% in Venezuela. Before fortification, folic acid deficit was present in 2.3% of school-age children, 24.5% of adults and 10.8% of the elderly population of the United States. Moreover, up to 61% of the Latin American and Caribbean population showed a reduced concentration of vitamin B12, which is caused by nutritional deficits affecting a large sector of the population, including vegetarians (29). Indeed, a report provided evidence of a low plasmatic level of vitamin B6 in the 40% of women from 21 to 44 years old (30). In a study conducted on 1,160 subjects (aged 67–96 years), Selhub et al. demonstrated that there is a strong inverse association between energy levels and concentrations of vitamin B6, B12, and folic acid. Similar inverse associations were demonstrated between homocysteine and intakes of folate and vitamin B6, but not vitamin B12. Prevalence of high homocysteine (>14 μmol/L) was found in 29.3% of subjects and was greatest among the subjects with low folate level (31).

Additionally, HCys levels tend to increase with age both in males and females and may vary according to different habits, such as cigarette smoking, alcohol consumption, and sedentary lifestyle (32, 33).

A reduction of HCys plasma levels has been linked to pregnancy (34).

### Cardiovascular Effects of Hyperhomocysteinemia

#### HHCys Effects in the Atherogenesis

**Figure 2:**
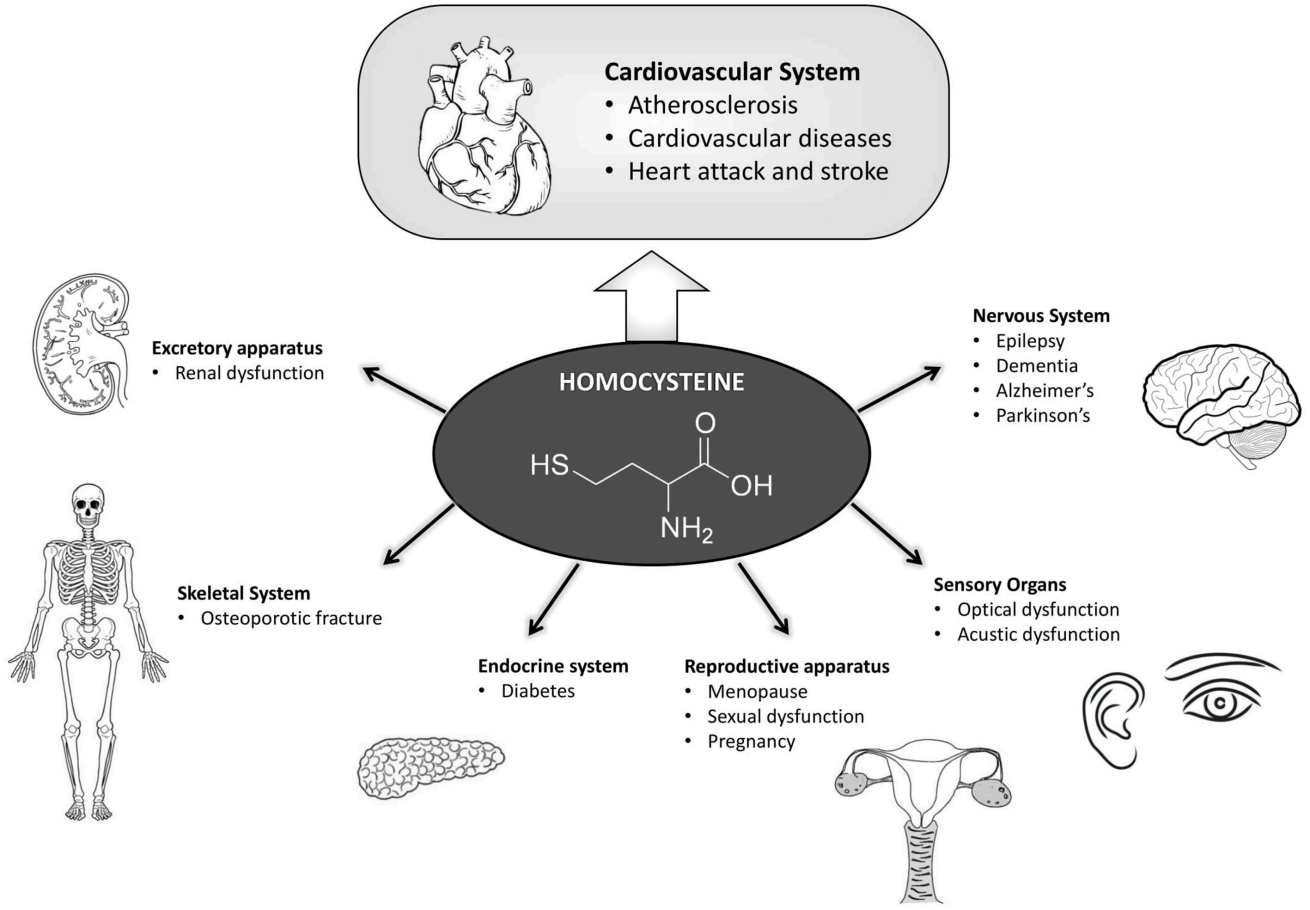
Scheme summarizing the pathologies in which hyperhomocysteine has a fundamental role in the onset and development. Hyperhomocysteinemia influences in a preponderant manner the cardiovascular system, which has been highlighted. Many other pathologies here reported are also affected by hyperhomocysteinemia due to their functional correlation with the cardiovascular system.

Many recent studies supported the theory of a correlation between increased levels of HCys and high blood pressure, both independent risk factors for cardiovascular disease and stroke (25, 35). An excess of HCys can favor atherogenesis, being therefore harmful for the cardiovascular system (Figure 2). Several studies correlated high level of HCys to cardiovascular diseases, for example retrospective case-control studies showed that 10% of the all coronary artery disease is attributable to high HCys levels or that an increase of the blood level of HCys at 5 μmol/l raises the risk of ischemic heart disease by 84% (36).

As an example, the accumulation of a HCys metabolite, the Homocysteine thiolactone (HTL), can be dangerous since, as a cyclic thioester with an esterified carboxyl group, it is highly reactive with the ε-NH_2_ groups of the lysinic residues of proteins (37), inducing protein homocysteinylation and thus producing changes in protein structure, activity and function (38, 39).

HHCys has several negative effects on the vascular system, including the alteration of endothelial function, which compromises the integrity of the vessel wall and in turn the vascular tone, leading to vascular inflammation (3, 40). HCys-induced endothelial dysfunction is caused by the reduction of nitrogen monoxide (NO), the most powerful vasodilator produced by the endothelium, and by the increase of oxidative stress following the production of reactive oxygen species (ROS) (3, 40). In addition, HCys alters lipid metabolism by initiating the process of oxidative degradation of endothelium membrane lipids, leading to the loss of function of cellular membranes (39).

HCys inhibits the production of NO in two ways, by the inhibition of NO synthase (eNOS) expression levels, supporting the over-expression of caveolin-1 that binds and inactivates eNOS (41, 42), or by reducing the cationic amino acid transporter that makes arginine available for the synthesis of NO by eNOS (43). Furthermore, HHCys causes an increase of asymmetric dimethylarginine levels, an endogenous eNOS inhibitor, probably by reducing the activity of dimethylarginine dimethylamminohydrolase, the enzyme that degrades asymmetric dimethylarginine (40, 44, 45).

Strong production of ROSs has been detected at the onset of HHCys when the sulfhydryl HCys group is easily oxidized, producing superoxide anion (${\text{O}}_{2}^{-}$) species as a result of both the activation of nicotinamide adenine dinucleotide phosphate oxidase and the inhibition of the expression and function of important antioxidant enzymes, such as superoxide dismutase and glutathione peroxidase (46). The superoxide anion (${\text{O}}_{2}^{-}$) easily interacts with NO forming peroxynitrite (ONOO^−^), an aggressive oxidizing reactive molecule, which further compromises endothelial function (44, 47). The increment of peroxynitrite (ONOO^−^) levels, induced by increased superoxide anion (${\text{O}}_{2}^{-}$) production, has been shown to cause in turn the formation of thromboxane A2 (TxA2), with a known arteriolar vasoconstrictive action, rather than prostaglandins (PGs) with vasodilatory action. Therefore, HHCys does not alter endothelial function only by inhibiting factors with vasodilatory action, but also by activating vasoconstriction factors (44).

HHCys can promote atherosclerotic lesion progression through endothelial dysfunction by increasing the expression of chemokines and adhesion molecules that induce a greater recruitment of circulating inflammatory blood cells (40). In fact, HCys favors the activation of the transcription factor NF-κB, which increases endothelial expression of monocyte chemoattractant protein-1 (MCP-1) and interleukin-8 (IL-8) (48). Moreover, HCys seems to favor monocyte proliferation and activation, leading to greater inflammatory cytokine production, while, at higher doses, it seems to reduce macrophage migration inhibitory factor (MIF) expression (49).

Furthermore, a high circulating concentration of HCys in the endothelium is demonstrated to be responsible for the increased proliferation of smooth muscle cells that, together with the alteration of normal platelet function, may contribute to the onset of atherosclerotic plaque formation. Endothelial cell exposure to high levels of HCys causes an increase of tissue factor expression, factor V, and factor XII activation (50), thus leading to thrombin formation through coagulation cascade propagation (40). In addition, the effect of HCys is enhanced by inactivation of the C protein, known as autoprothrombin II2A and blood coagulation factor XIV, as well as by the inhibition of the fibrinolytic process via a reduction in the activity of the tissue plasminogen activator and an increase in the plasminogen activator inhibitor (51).

Furthermore, in endothelial cells HCys can increase the expression level of chemokines, adhesion molecules (VCAM-1), tissue factor, RAGE, MMP-9, and chemo-attractant proteins like MCP-1 and IL-8, leading to the activation of chemo-taxis activity in human peripheral blood monocytes.

Ultimately, HHCys can induce apoptosis of endothelial cells through the activation of the Fas cell-death pathway, the p53/Noxa pathway, and the cytochrome-c/Cas-3 and 9 pathways. Recently, it has been reported that HCys upregulates platelet-derived growth factor levels by DNA de-methylation, affecting the cross-talk between endothelial cells and vascular smooth muscle cells (52).

#### Genetic Variation of the MTHFR Gene and Cardiovascular Diseases Risk

A moderate increase of the HCys levels over a lifetime appears to have little effect on coronary heart disease, whereas a common genetic variation of the MTHFR gene (*C677T*) strongly influences the levels of the HCys and cardiovascular risk (53). In particular, subjects with the TT genotype of the *C677T* polymorphism show HCys levels 20% higher than those with the CC genotype. The heterozygous CT genotype has an intermediate effect, but is closer to the TT genotype than CC (54).

Interestingly, a meta-analysis study on HCys blood levels highlighted that a 25% reduction in HCys is associated with a significant reduction of ischemic heart disease, while an increment of 5 μmol/l is associated with a 32% increase of ischemia and with a 59% increased risk of stroke (3). Moreover, HHCys has been confirmed as a coronary artery disease risk factor, with a well-defined prognostic value: the plasma total levels of HCysis are a strong predictor of mortality in patients with coronary artery disease diagnosed angiographically (55). As shown in a meta-analysis study, HHCys can also be considered an aortic aneurysm-inducing factor, in fact circulating HCys levels have been correlated with abdominal aortic aneurysm (AAA) (56). Conclusively, increased HCys levels are also involved in generalized small-vessel disease (CSVD), as confirmed by a SMART-MR (Second Manifestations of ARTerial disease-Magnetic Resonance) study (57).

Administration of folic acid, Vitamin E and vitamins B6, and B12 in addiction to betaine reduces the risk of thrombosis and cardiovascular dysfunction in patients with severe HHCys. Additionally, folic acid therapy entails determinants in the degree of lipid peroxidation, platelet activation and their downregulation (53). Therefore, daily supplementation with folic acid has been shown to lower the plasma HCys level by approximately 25%, and adding vitamin B12 further lowers the level by approximately 7%, indicating the pivotal role of vitamin B supplements in significantly lowering HCys levels (58). Furthermore, HCys levels increase over time in heart transplant recipients, and folate supplementation appears to reduce HHCys in heart transplant patients (59).

Furthermore, vitamin B2 administration reduced hypertension induced by HHCys and, interestingly, it has been shown that in patients homozygous for the *C677T* polymorphism of MTHFR the intake of vitamin B2 reduces blood pressure in the TT genotype—not only in other hypertensive patients. Genotype TT is associated with hypertension, and these patients are more resistant to antihypertensive treatment (22).

#### Hyperhomocysteinemia and Stroke

HHCys is also considered an independent risk factor for peripheral vascular diseases such as stroke (35, 60) (Figure 2). Although little is known about HCys plasmatic modifications in the acute phase of cerebrovascular diseases, a significant increase of homocysteine blood level in patients with stroke has been observed, which was enough to be considered as a possible marker for the acute phase rather than a risk factor for ischemic events.

Evidence has shown that the genetic polymorphism of cystathionine β synthase (CBS) *T833C*, an enzyme involved in HHCys, was associated with an increased risk for developing stroke (24).

Recently, a meta-analysis study demonstrated the efficacy of folic acid supplementation in stroke prevention, also combined with statins therapy; moreover, folic acid supplementation has proven synergic activity, potentiating the anti-hypertensive effect of the Enalapril drug, in turn reducing stroke risk (9).

Vitamin B (cofactor for MTHFR) modulates the HCys level of patients carrying MTHFR *C677T* polymorphism; in populations with low folate consumption compared with geographical areas showing higher dietary folate intake, this polymorphism was associated with a larger effect on HCys concentration, so that a clear association between this genetic variant and stroke risk was therefore established (61). Interestingly, the consumption of folic acid combined with B vitamins demonstrated a potential benefit in primary stroke prevention, especially in males (62). Thus, high HCys level in the acute phase of stroke was not associated with stroke severity but with a higher risk of small artery disease subtype of stroke (63).

An interesting study has shown that the usage of nutraceutical compositions can reduce the risk of cardiovascular pathologies in subjects presenting HHCys and hypertension. The authors enrolled patients affected by essential hypertension and HHCys, and they were split into two groups: one group received a combined nutraceutical containing folate-6-5-methyltetrahydrofolate, vitamin B6, vitamin B12, vitamin B2, zinc, and betaine (Normocis^400^^®^) once daily for 2 months, while the other group received a conventional supplementation with highly dosed folic acid (64). Notably, Normocis^400^^®^ was more efficient in reducing the HCys blood level than folic acid alone, meaning that a combined and well-balanced nutraceutical composition can actively protect body cells from HHCys, giving evident therapeutic support against cardiovascular and associated diseases.

### Effects of the Hyperhomocysteinemia in the Nervous System

HHCys has an important role in the pathogenesis of various diseases affecting the nervous system, such as stroke, Parkinson's disease, Alzheimer's disease, multiple sclerosis, epilepsy, etc. (13), although its molecular mechanism in this role is not yet fully defined (Figure 2).

Specifically, HCys is an amino acid with excitatory activity that can became toxic for both murine and human neurons (13). Alterations of glutamatergic transmission can lead to the toxic condition called “*excitotoxicity*,” in which the hyperactivity of glutamatergic receptors that causes changes in intracellular calcium homeostasis is involved in the development of numerous neurological diseases (65). Interestingly, HCys proved to have agonist and partial antagonist activity on NMDA glutamate receptors, respectively, on the glutamate and glycine binding sites (66).

As previously described, high HCys levels cause increased ROS production which, besides the negative cardiovascular effects, negatively influences the brain, inducing neuronal damage, and leading to neuronal cell death (13). Indeed, it has been reported that HCys promotes mitochondrial dysfunction through Cu^2+^ chelation, which results in cytochrome C oxidase inactivation (67).

Other mechanisms that associate HHCys effects to cell damage in the nervous system are related to inflammatory processes (13, 68). Parallel to the effect on endothelial cells, HCys is able to increase NF-kB expression and activity also in the nervous system. In fact, an induced high level of HCys was shown to increase NF-kB levels in a neuroblastoma cell line, an effect prevented by the administration of antioxidants (69). Moreover, HCys administration brought to an augmented level of several pro-apoptotic markers, such as Bax, p53, and caspase-3, suggesting a correlation between HCys-induced cell damage and NF-kB activation (69).

On the other hand, according to some studies, HHCys may be the consequence of immune system activation rather than the cause. In fact, the increase of ROS production, induced by immune system activation, involves a greater demand for antioxidants, such as vitamin B12 and folate, and in case of a non-sufficient dietary intake this could lead to HHCys (13, 68).

#### Hyperhomocysteinemia in Cognitive Impairments, Alzheimer's, Parkinson's Disease and Epilepsy

HHCys has been related to Alzheimer's and Parkinson diseases (70) (Figure 2), particularly in the late stages of the illnesses or after long-term levodopa treatment (71). As an example, a longitudinal study, lasting ≥8 years, including 1,092 individuals with dementia (mean age = 76), showed that the risk of developing AD was doubled in patients with levels of HCys > 14 μmol/l (72). Mildly elevated HCys levels may also increase the risk of developing non-AD dementia (73), vascular dementia, Parkinson's disease-associated dementia, and multiple sclerosis-associated cognitive decline (74).

Moreover, patients with epilepsy exhibit elevated plasma HCys level more frequently than the general population (10–40% vs. 5%) due to reduced activity of the MTHFR enzyme caused by polymorphisms on its gene (8).

The mechanism by which levodopa treatment induces HHCys has been largely studied and it was demonstrated that it is linked to catechol-O-methyltransferase (COMT) activity, since COMT inhibitors (COMT-I) are capable of reducing HCys levels (75). The ability of COMT-I to reduce or prevent levodopa-induced HHCys in Parkinson's disease patients may be attributed to differences of their vitamin status. In patients with low or low-normal folate levels, levodopa administration increases HCys level, while the administration of a COMT antagonist, such as entacapone, induces a great reduction (76). Notably, the lifestyle of Parkinson's disease patients treated with levodopa seems to be very important to determine the plasma level of HCys. Indeed, coffee consumption, smoking and alcohol use are positively associated with high HCys plasma levels. In any case, the MTHFR *C677TT* polymorphism remains the major determinant of coffee-induced HHCys (7).

Folic acid and vitamin B2, B6, and B12 supplementation reduced levodopa-induced HHCys, and high-dose folic acid and vitamin B6 and B12 consumption slowed the rate of accelerated brain atrophy in subjects affected by mild cognitive impairment (6).

Clinical studies have demonstrated the association between risk of cognitive decline and high HCys plasma levels (77) together with a correlation between HHCys and cognitive Alzheimer's impairment. Indeed, vitamin intake, including vitamin B12/B6 and folic acid, partially counteracted cognitive damage (72). Moreover, an association between HHCys and depression has been found, as the lack of folate, vitamin B12, and vitamin B6 levels seems to be related with depression. The intake of vitamin B12, vitamin B6, and folate in depressed subjects can consistently improve cognitive performance and decrease the level of total HCys (78). In addition, it was demonstrated that folic acid supplementation over a 3 year period in men and postmenopausal women aged from 50 to 70 years reduced the rate of cognitive decline (79).

Elevated total plasma HCys has been established as an independent risk factor for epilepsy. Anti-epileptic drugs (AED) like carbamazepine, phenobarbital, primidone, and phenytoin are associated with the reduced serum levels of folate and vitamin B12, and this may be mediated by AED side effects (80). Around 20–40% of epileptic patients exhibit ultra-physiological plasma levels of HCys as a consequence of the interplay between variants of the MTHFR gene polymorphisms. Folic acid alone or combined with other B-vitamins has the potential to reduce HCys concentration in patients under chronic treatment with antiepileptic drugs (8, 81).

### Hyperhomocysteinemia and Pregnancy, Menopause, and Sexual Dysfunction

Elevated plasma levels of HCys during pregnancy are associated with placental vascular damage that may be correlated with abortion, preeclampsia or other unfavorable outcomes of gestation such as pregnancy diabetes (82) (Figure 2). Interestingly, HCys plasma levels are lower in women within reproductive age compared to men of the same age, although it increases after postmenopausal age, thus explaining the higher risk of cardiovascular disease documented in postmenopausal women (83). It was observed that the administration of low doses of folic acid and vitamin B12 reduced HCys plasma levels after few months of treatment, suggesting their potential cardio-protective effects (84). HCys plasma level was found to be a predictive marker for pregnancy-induced hypertension (PIH) (85), and HHCys was also associated with an increased risk of puerperal cerebral venous thrombosis (CVT) (86). As an example, in an Indian case-control study, the adjusted odds ratio for the risk of puerperal CVT with hyper-Hcy (>90th percentile) was 10.8 [95% CI: 4.0–29.4; adjusted for vitamin B (12) and folate levels]. Low folate and vitamin B (12) levels (< 10th percentile) did not increase the risk for puerperal CVT. There was a significant inverse correlation between folate and HCys levels (rho = −0.471, *p* < 0.001) (86).

In another Indian case-control study, homocysteine was found as high as 93.75 % in patients affected by CVT (85).

Folate deficiency and HHCys are associated with Neural Tube Defects (NTDs) and other fetal abnormalities such as schistorrhachis (87). As a matter of fact, a high frequency of MTHFR polymorphisms has been found in mothers with fetuses affected by neural tube defects and cardiac malformations. However, a case-control study demonstrated that genetic polymorphisms played only a small role in NTDs, and consuming folic acid, vitamin B12 and B6 appears to reduce these congenital malformations (88). Vitamin B6 also counteracted nausea and vomiting in early pregnancy (89).

Folate supplements reduced the risk of delivering newborns affected by autism spectrum disorders (ASD) (90) and improved children's language competency (91), although high dosages of folic acid supplements during pregnancy proved to have negative consequences on psychomotor development after the first year (92). An elevated plasma HCys level in early pregnancy can increase the risk of developing severe preeclampsia (93), and supplementation of multivitamins containing folic acid in the second trimester is associated with its reduction (94). Furthermore, HHCys was inversely related to insulin sensitivity in preeclampsia (95). When the plasma HCys level rises >10 μmol/L in early pregnancy, the risk of developing severe preeclampsia has been shown to be 51.3% (93).

Folate deficiency and HHCys are also important for oocyte quality and maturation, implantation, placentation, fetal growth, and organ development, correlating with subfertility (96).

Unfortunately, there is a still an insufficient number of women taking folic acid food supplements during their reproductive age, and rarely does the diet have sufficient folate content, which is the reason why the intake of folate food supplements is recommended (97). Recent data demonstrated that micronutrients and vitamin supplementation in pregnancy reduced morbidity and immune function of infants during the first 6 months of life (98).

Moreover, folate deficiency in postmenopausal women is linked to cognitive impairments and dementia of the newborn (99). Indeed, a progressive decrement of the HCys blood levels to 1 μmol/L is associated with approximately a 10% of vascular disease risk reduction while the risk diminush of 30–40% if the blood level of HCys is reduced of 3–4 μmol/L. HCys level was found to be affected by sex hormone concentrations; indeed, males have increased levels compared to females of the same age. Furthermore, postmenopausal women have higher concentrations compared to premenopausal women. The increased risk of cardiovascular disease documented in postmenopausal women is related to the increase of HCys levels, and low-dose folic acid administration induces the reduction of HCys plasma levels of the same degree observed for hormone therapy (83).

Interestingly, erectile dysfunction (ED) is also associated with vascular damage, therefore HHCys has been related to erection disorder (100) (Figure 2).

### Homocysteine Levels and the Risk of Osteoporotic Fracture

Osteoporosis is characterized by a low density of mineral in bones accompanied by deterioration of bone microarchitecture and an increased risk of fracture. Osteoporotic fractures are associated with increased morbidity and mortality (101, 102). It has been hypothesized that the metabolism of HCys is involved in osteoporosis (Figure 2). Interestingly, the relationship between circulating levels of HCys and the incidence of fracture has been investigated in two independent prospective studies of three groups of men and women of 55 years of age or older. The observed association between HCys levels and the risk of fracture was primarily associated with bone mineral density and also with dietary intake of calories, protein, calcium and vitamins (103). For example, an association between circulating HCys levels and the risk of incident osteoporotic fracture has been shown in 2,406 subjects >55 years of age (RR = 1.4 per 1 SD increase in the natural-log–transformed homocysteine level, 95 % CI, 1.2–1.6) (103), while the elevations of HCys (>20 μmol/L for men and >18 μmol/L for woman) confer a sizeable risk increase for bone fracture (4.1-fold men, 1.9-fold for women) (104).

### Hyperhomocysteinemia and Autoimmune Rheumatic Disease

Rheumatic diseases are frequently associated with a high prevalence of coronary events (105), indeed patients with rheumatoid arthritis (AR) and systemic lupus erythematosus (LES) (Figure 2) develop precocious atherosclerosis and show increased mortality (106).

A case-control study showed that patients affected by AR have HHCys in respect to controls (17.3 ± 7.8 vs. 7.6 ± 1.9; *p* < 0.001) (107), while it has been reported that patients with SLE have a prevalence of myocardial infarcts in ranges from 4 to 45% correlated with their HHCys (108).

HHCys was considered as a risk factor for cardiovascular disease both in AR and LES patients. As was shown before, the increase of HCys level has a direct toxic action on endothelial cells and promotes LDL oxidation and prothrombotic effects. Methotrexate is the elective drug for the treatment AR, but, being a methylenetetrahydrofolate reductase inhibitor, it reduces the plasma and erythrocyte levels of folate, thereby causing an increase of HCys levels that indeed become dangerous for healthy cells, as well. Therefore, the strategy suggested by a recent study was to administer methotrexate in conjunction with folic acid supplementation (108).

### Hyperhomocysteinemia and Diabetes

The studies conducted on diabetic patients did not provide unique results on the relationship between plasma HCys level and diabetes (Figure 2). Acute hyperinsulinemia type-1 is associated with the reduction of HCys levels in control subjects, but not in type-2 diabetes, indicating that insulin could differently regulate HCys metabolism. Indeed, recent data demonstrated a correlation of HHCys with diabetes complications since patients affected by diabetic angiopathy showed high homocysteinemia levels. This correlation is dependent on the glomerular filtration rate that regulates HCys level (109). Moreover, patients with microalbuminuria and proliferating retinopathy showed homocysteinemia values significantly higher than those of patients without such complications; however, it does not seem to be mediated by confounding factors or by differences in the vitamin balance. Furthermore, recent data show that folic acid supplementation may reduce HCys levels in patients with type-2 diabetes mellitus and produce a better glycemic control compared to placebo (110).

### Hyperhomocysteinemia and Renal Dysfunctions

As reported above, HHCys is related to the glomerular filtration rate, therefore when renal function declines HCys values increase and progress to uremia (filtrate glomerular falls below 70 ml/min) (Figure 2). In uremia conditions, the accumulation of AdoHCys, precursor of HCys, is caused by an inhibition trans-methylation reactions (111), which consequently produces a lack of protein methylation, DNA hypomethylation and alteration of the allelic expression of genes regulated by methylation.

The increase of the HCys plasma level reported during chronic renal failure (CRF) induces premature cardiovascular disease (112). In one case-controlled study, 85% of hemodialysis patients showed HCys levels above the 95th percentile for normal controls; and HHCys also significantly increased the risk for vascular comorbidities (OR = 2.9; 95% CI, 1.4–5.8) (113).

In fact, the dysfunction of kidney or other organs induces the accumulation of increasing amounts of HCys, which is associated with cardiovascular events in CRF patients (114). A meta-analysis study suggests that daily folic acid oral administration (115, 116) or intravenous injection (117) promote HCys reduction in subjects with normal renal function, but not in hemodialysis patients. Furthermore, recent data demonstrated that with intravenous administration of low doses of folic acid and vitamin B12 the plasma levels of HCys are significantly reduced (118).

### Hyperhomocysteinemia in Acoustic and Optical Dysfunctions

Sudden hearing loss (SHL) is a mono or bilateral inner dysfunction characterized by the loss of 30 dB on three frequencies contiguously manifesting in a few minutes or hours (Figure 2). The acoustic damage, detected by using auditory brainstem responses (ABRs), was shown to be accompanied by a high dosage of HCys in the blood. In line with the fact that HHCys is a thrombotic risk factor, SHL, and vestibular damage are also known as “cochlea ischemia” because of the cochlear vascularization failure that is detected in these pathologies. A recent study demonstrated that SHL is associated with MTHFR gene polymorphisms (*C677T* and *A1298C*) which are also associated with high HCys blood levels and low levels of folate (119).

Several optical diseases are characterized by cardiovascular alterations, which lead to retinal degradation and loss of sight. Recent studies have demonstrated that HHCys is a risk factor for age-related macular degeneration (AMD). AMD is the leading cause of severe irreversible vision loss in older patients, and case-report studies demonstrated that vitamin B12/B6 and folic acid administration on women with an increase of cardiovascular risk decreased ADM onset (120). A study showed that 59 patients affected by the neovascular AMD had 27.9% higher levels of HCys than the group of patients with dry AMD, and 21.9% higher levels than the control group (*P* < 0.02). Hyperhomocysteinemia was found in 44.1% of the studied group, in 22.4% of the dry AMD group, and in 21.4% of the control group (*P* = 0.011) (121).

### Hyperhomocysteinemia, Migraine, and Headache

HHCys may contribute to the alteration of cerebral flow, with risk of thrombosis and alteration of the cerebral oxygen transport, ultimately promoting migraine aura events (122) (Figure 2). Recent studies have demonstrated that the *C677T* polymorphism on the MTHFR gene can influence the susceptibility to migraine, with the migraine being due, as reported above, to the increase of HCys levels in the blood (123).

### Hyperhomocysteinemia and Psoriasis

Psoriasis is a chronic inflammatory disease of the skin that affects the 3% of the population. It is characterized by erythema-squamous plaques distributed symmetrically in typical sites (elbows and knees, sacral region and scalp). Several studies demonstrated that psoriasis was related to increased mortality for coronary heart disease (124), cerebrovascular disease and pulmonary embolism (125). Other studies have shown that psoriasis was connected to subclinical vascular alterations like intima-media thickness, arterial wall stiffness and coronary calcifications, confirming the tight association between atherosclerosis and psoriasis (126).

Interestingly, psoriatic patients showed an increased risk of developing atherothrombotic diseases, and in parallel, psoriasis duration and severity seem also to be correlated with an increased cardiovascular risk, although biomarkers are still missing. In line with such an interaction between psoriasis and cardiovascular disease, classical systemic anti-psoriatic therapy (cyclosporine, retinoids, and methotrexate) can increase the risk factor for cardiovascular attack together with an increased homocysteine expression level (127) (Figure 2).

Furthermore, HHCys seemed to be associated with psoriatic disorder and with platelet hyperactivity that promotes prothrombotic events, which determine increased risk of death caused by arterial and venous thrombosis. Dietary modifications appear relevant in helping the global management of patients with moderate to severe psoriasis (128). However, in a study on psoriatic patients, the decrease in folic acid level was more attributed to an increased consumption from proliferating keratinocytes rather than to the correlation between the serum folate level and the duration or extent of clinical psoriasis (129). Plasma HCys directly correlated with psoriasis severity according to skin psoriasis area and severity index (PASI) score (the clinical gold standard measure of psoriasis severity), whereas it was inversely correlated with plasma folic acid levels, which were lower in psoriasis patients than in the controls and lower than the normal range in 13 of 40 (32.5%) psoriasis patients studied (127).

## Discussion

This review summarizes several observations regarding possible correlations between HHCys and diverse pathologies when a dysmetabolism of HCys occurs in the involved cells (Figure 2). Today, the literature shows several studies in which there seems clearly to be a common correlation between the metabolism of methionine and homocysteine and their influences in several pathologies.

The metabolism and catabolism of methionine and homocysteine are based on complicated biochemical pathways that involve the cooperation of several enzymes and that go through the production of different molecules that represent fundamental biochemical steps for cell survival.

As we here report, the disequilibrium of methionine metabolism leads to hyper-production and accumulation of homocysteine, a condition known as HHCys which, in line with present scientific literature, seems to be a risk factor for cardiological diseases, as well as for other conditions here listed.

Interestingly, HHCys is currently not only seen as the diagnostic marker for pathologies but it is also considered a possible therapeutic target. It has been reported that a diet deficient in folic acid, vitamin B6, vitamin B12, and betaine is responsible for the development of HHCys. Consequently, being able to compensate the deficits of these important components must be considered of high therapeutic relevance in clinical practice.

Several studies have reported that the administration of folate, group B vitamins and other molecules that enter into the metabolic cycle of methionine is able to decrease the severity of HHCys, being helpful in several pathological conditions and also in pregnancy.

This review is intended to be a summary of several pieces of evidence that can show how HHCys is implicated in several pathologies, and although it is far from being considered a biomarker of these pathologies, it might still be a good target for clinical interventions.

## Author Contributions

CT, AD, and EF wrote the manuscript. SM read and reviewed the manuscript. MF supervised, read, and reviewed the writing, seeked fundings.

### Conflict of Interest Statement

The authors declare that this study received funding from INPHA2000 srl to pay the publication fee. The funder had no role in study design, data collection and analysis, decision to publish, or preparation of the manuscript.
